# Effects of soy milk ingestion immediately after resistance training on muscular-related biomarkers in older men: a randomized controlled trial

**DOI:** 10.5114/biolsport.2023.123894

**Published:** 2023-06-15

**Authors:** Babak Hooshmand-Moghadam, Monika Johne, Fateme Golestani, Katarzyna Lorenz, Monireh Asadi, Ewelina Maculewicz, Andrzej Mastalerz

**Affiliations:** 1 Department of Exercise Physiology, Ferdowsi University of Mashhad, Mashhad, Iran; 2 Department of Biomedical Sciences, Józef Piłsudski University of Physical Education, Warsaw, Poland; 3 Department of Exercise Physiology, University of Birjand, Birjand, Iran; 4 Faculty of Sports Sciences, Ferdowsi University of Mashhad, Mashhad, Iran

**Keywords:** Aging, Soy milk, Hypertrophy, Skeletal muscle, Resistance training

## Abstract

We evaluated the effects of soy milk ingestion on changes in body composition, strength, power, and muscular-related biomarkers following 12 weeks of resistance training in older men. Thirty healthy older men (age = 65.63 ± 3.16 years; body mass = 62.63 ± 3.86 kg) were randomly assigned to one of two groups: soy milk + resistance training (SR) or placebo + resistance training (PR). Participants in the SR group received 240 ml of vanilla-flavoured non-dairy soy milk immediately after every training session and at the same time on non-training days. Differences in muscle mass, upper limb body strength (UBS), lower limb aerobic power (LAP), activin A, and GDF15 were significantly greater in the SR group vs. the PR group (P < 0.05). Both intervention groups experienced a significant (p < 0.05) reduction in body mass (PR = -3.9 kg; SR = -3.2 kg), body fat % (PR = -0.8%; SR = -1.2%), activin A (PR = -5.1 pg/ml; SR = -12.8 pg/ml), GDF15 (PR = -8.1 pg/ml; SR = -14.7 pg/ml), TGFβ1 (PR = -0.43 pg/ml; SR = -0.41 pg/ml), and increase in muscle mass (PR = 0.81 kg; SR = 2.5 kg), UBS (PR = 3.4 kg; SR = 6.7 kg), lower limb body strength (PR = 2.8 kg; SR = 5.2 kg), upper limb aerobic power (PR = 34.3 W; SR = 38.6 W), LAP (PR = 23.2 W; SR = 45.2 W), BDNF (PR = 8.3 ng/ml; SR = 12.7 ng/ml), and irisin (PR = 1.5 ng/ml; SR = 2.9 ng/ml) compared to baseline. The ingestion of soy milk during 12 weeks of resistance training augmented lean mass, strength, and power, and altered serum concentrations of skeletal muscle regulatory markers in older men.

## INTRODUCTION

Aging is the age-related decline of biological functions [1]. Aging is a natural process associated with modifications in biological systems such as a decline in muscle mass and muscle strength [2]. Skeletal muscle accounts for approximately 40% of total body mass, and it plays an indispensable role in locomotion and metabolism [3, 4]. Between the ages of 20 and 80, humans lose approximately 20–30% of their skeletal muscle mass. Skeletal muscle undergoes a gradual loss of fat-free mass, size, and function in the aging process called sarcopenia [2]. Several possible mechanisms for age-related muscle atrophy have been described; however, the precise contribution of each is not well known. Age-related muscle loss is a result of reductions in the size and number of muscle fibres, possibly due to a multifactorial process that involves physical activity, nutritional intake, oxidative stress, and hormones or signalling molecules [5]. It has been suggested that elevations in circulating concentrations of cell-signalling molecules increase the likelihood of receptor interaction and thus enhance the probability of a physiological effect within skeletal muscle [6].

Muscle mass is determined by the balance between muscle protein synthesis (MPS) and muscle protein breakdown (MPB) [5]. The main anabolic pathways that lead to protein synthesis in muscle include serine/threonine kinase, protein kinase B, mammalian target of rapamycin (mTOR), hormones such as insulin-like growth factor and insulin and branched-chain amino acids (e.g. leucine, valine, isoleucine) [7]. Muscle protein breakdown includes activation of the ubiquitin-proteasome pathway by changing transforming growth factor-beta (TGF-β) and myostatin [7].

Tumour growth factor-β1, a member of the TGF-β family, is a multifunctional cytokine involved in the regulation of muscle repair via satellite cell activation, connective tissue formation, and regulation of the immune response intensity [8, 9]. Growth and differentiation factor-15 (GDF15) has both environment/situation mediated and direct effects on skeletal muscles, resulting in reduced muscle mass, and therefore it is potentially involved in sarcopenia [8, 10]. Baseline GDF-15 predicts declining physical activity in the elderly [11]. Irisin is a novel myokine that has been shown to induce browning of white adipocytes. Irisin improves myogenesis and induces hypertrophy of skeletal muscle since it is secreted from muscles in response to exercise [12, 13]. Brain-derived neurotrophic factor (BDNF) as a protein is produced in the skeletal muscle and increased by muscle contraction to enhance fat oxidation, which can regulate glucose and fat metabolism [14]. Lower concentrations of BDNF and irisin, and higher concentrations of activin A, growth and differentiation factor-15 (GDF15), and tumour growth factor β1 (TGFβ1) have been reported during aging [15–18].

Resistance training improves musculoskeletal strength, muscle mass, bone mass, and connective tissue thickness [19]. Previous research has shown that strength training increases power and strength, muscle size, and plasma-metabolism biomarkers in the elderly [2, 15, 20–22]. Resistance training is a potent stimulus to increase skeletal muscle mass and results in an anabolic response [23]. The muscle protein accretion process depends on a robust synergistic action between protein intake and overload [23]. It is suggested that post-exercise protein ingestion increases plasma amino acids, which results in the activation of signalling molecules, leading to an increase of MPS and muscle hypertrophy, particularly leucine, thus activating the key anabolic signalling mechanism of the mechanistic target of rapamycin (mTOR) [23–25]. The balance between MPS and MPB is dependent on protein consumption and the accompanying hyperaminoacidaemia, which stimulates a marked rise in MPS and mild suppression of MPB [26]. Recently, it was suggested that soy milk (a plant-based non-dairy beverage) can be considered to be a nutritionally adequate and complete protein, which is enriched in fatty acids, phosphatidylcholine, and isoflavones, particularly genistein [27]. Soy milk is a good source of protein, calcium, and potassium [28]. Also, it has been reported that soy milk contains essential amino acids such as leucine, lysine, phenylalanine, and isoleucine [29, 30]. Based on the authors’ knowledge, the present study is the first one to evaluate the effects of soy milk consumption and resistance training on serum concentrations of activin, GDF15, TGFβ1, BDNF, and irisin in older men. However, there have been limited studies with contradictory findings that have been conducted to examine the effects of soy milk consumption and resistance training on body composition, strength, and power. Therefore, we conducted the present study to assess the effects of soy milk ingestion on changes in body composition, strength, power, and muscular-related biomarkers (activin A, GDF15, TGFβ1, BDNF, and irisin) following 12 weeks of resistance training in older men.

## MATERIALS AND METHODS

### Participants

**FIG. 1:**
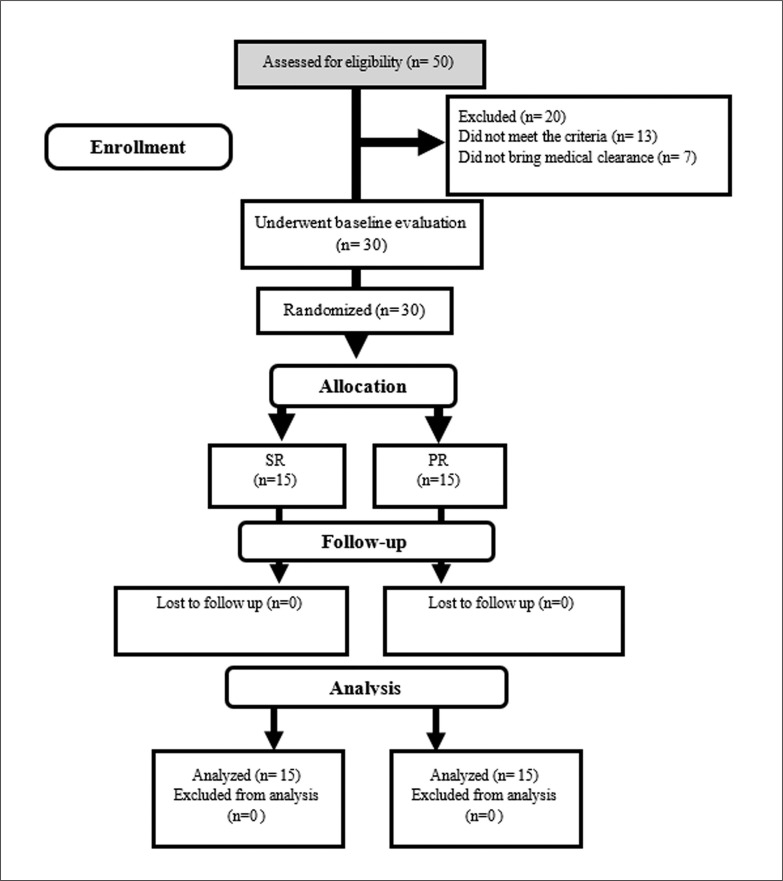
Participants’ flow diagram.

Thirty healthy elderly men (age 65.63 ± 3.16 years; body weight = 62.97 ± 4.18 kg) volunteered for this study (Figure 1). Age 60–80 and physical independence were inclusion criteria. Exclusion criteria were: current or previous significant cardiovascular histories; neurological, respiratory, muscular, metabolic, inflammatory, bone, joint, and movement disorders; consumption of dietary supplements; alcohol consumption or smoking for at least 1 year prior to study entry; allergy/sensitivity to soy milk and a history of regular physical activity in at least the last year. All of these criteria were assessed by the physician using the Physical Activity Readiness Questionnaire (PAR-Q) and the Health/Medical History Questionnaire. Written informed consent was obtained from all participants. All experiments were performed in accordance with the Declaration of Helsinki. This study has been approved by the Sport Sciences Research Institute of Iran (IR.SSRC.REC.1398.062) and registered in the Iranian Clinical Trials Registry (IRCT20190731044398N3).

### Study design

**FIG. 2:**
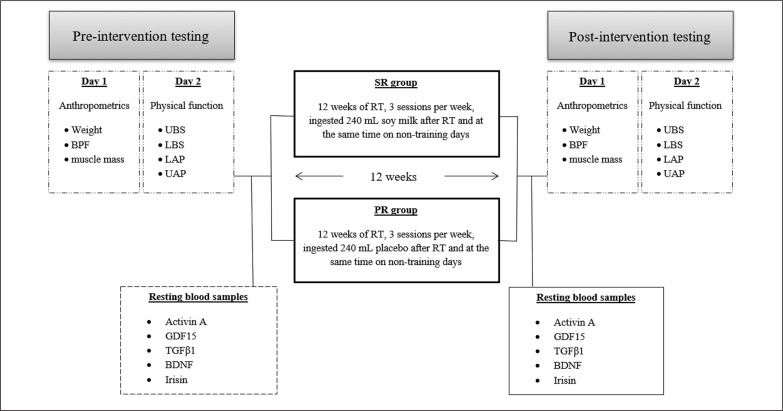
Diagram of the study design. Abbreviations; SR: soy milk + resistance training; PR: placebo + resistance training; BMI: Body Mass Index; BFP: Body Fat Percent; UBS: Upper body Strength; LBS: Lower body Strength; LAP: Lower Anaerobic Power; UAP: Upper Anaerobic Power.

This study was a randomized, double-blind, parallel prospective clinical trial (Figure 2). Prior to baseline measurements, all participants were familiarized with all tests and procedures. The participants were then randomized to two groups: soy milk + resistance training (SR; n = 15) or placebo + resistance training (PR; n = 15). The allocation was stratified using a digital tool available at www.randomizer.org. Measurements were collected at the beginning and end of the 12 weeks of intervention. All measurements were recorded at the same time and under the same environmental conditions. Participants were asked not to change their current lifestyle and eating habits during the study period.

### Interventions

#### Supplementation protocol

Participants in the SR group received 240 ml of vanilla-flavoured dairy-free soy milk immediately after each training session and simultaneously on non-training days for 12 weeks. Also, participants in the PR group received 240 ml of placebo (a placebo with artificially sweetened water) at the same time as the SR group. Soy milk and placebo were in opaque (masked) beverage containers and were similar in taste and smell, texture and appearance [31]. The timing and dose of soy milk were chosen according to the previous studies conducted by other scientists [30, 32]. Soy milk (dairy-, lactose-, and casein-free; absolutely no carrageenan, gluten, egg, or peanut) was supplied from Saina Ghaza Part Company, Iran and registered with the health ministry (No:56/16554). The energy and macronutrient composition of this milk per 240 mL are as follows: energy, 99.6 kcal; protein, 6.75 g; carbohydrate, 9.15 g; fat, 4 g; calcium, 100 mg; sodium, 98 mg; cholesterol, 0 mg; iron, 1.1 mg; vitamin D 2.5 mcg; vitamin A, 150 mcg; vitamin B12, 1.5 mcg; potassium, 300 mg; riboflavin 0.4 mg. It should be noted that researchers monitored the soy milk supplementation. After the training sessions, soy milk was served to the participants by the researcher, who witnessed its consumption. On non-training days, supplementation was verified by phone or SMS. To assess compliance with supplementation on non-training days, participants provided empty nutrient boxes to study staff.

#### Exercise training programme

In order to familiarize themselves with the training procedures, the members of the two groups were familiarized with the correct form of lifting and correct breathing techniques. Participants in these groups performed sessions of the resistance training programme on three non-consecutive days per week for 12 weeks. The training programme consisted of three stages: a 10-minute warm-up, 40-minute resistance training and a 10-minute cool-down (Table 1 shows the details of the training programme). The resistance training (four sets/six exercises/15 tempo-controlled repetitions/30-second rest between each exercise) included leg presses, chest presses, rows, leg stretches, pull-ups, and triceps push-ups. Participants began training at ~60% of one repetition maximum (1RM) at the beginning of the intervention; if they could comfortably do the exercise for about 60 seconds, we added about 5% weight gain for the next training session. 1RM was determined as described by Calvani et al. [33]. The periodized protocols were adapted from previous studies [34]. All training sessions were supervised by qualified personal trainers.

**TABLE 1:** Resistance training program

SR and PR Groups	
Sessions per week	3
Warm-up	10 min treadmill running/ low density
Resistance training	**Exercise:** Leg Press, Chest Press, Seated Row, Leg Extension, Lat Pulldown, Tricep Dips **Intensity:** 60% 1RM **Volume:** 1 min per exercise, 30 sec recovery per exercise **Repetition:** Repeat × 4 sets
Cool-down	10 min

Abbreviation: SR: soy milk + resistance training; PR: placebo + resistance training.

### Measurements

#### Body composition assessments

Upon arrival at the laboratory, participants were asked to urinate completely within 30 minutes of the test. Body weight (BM) was measured using a digital scale (SECA, Germany) with an accuracy of 0.1 kg. The height of the participant was measured with a stadiometer (SECA, Germany) with an accuracy of 0.1 cm. Also, body fat percentage (BFP), body mass index (BMI), and muscle mass were assessed using a multi-frequency bioelectrical impedance device (BIA; Jawon Medical X Contact-356, South Korea), as previously described [35]. The test-retest reliability of the bioelectrical impedance method is high (R = 0.95 to 0.99) [36].

### Physical performance tests

Lower limb anaerobic power (LAP) and upper limb anaerobic power (UAP) were evaluated by the Wingate anaerobic (Monark 831E and 894 Ea, Varberg, Sweden) test as previously described [36]. A medical doctor supervised the Wingate testing to monitor signs of cardiovascular discomfort. VO_2max_ of each participant was measured by the modified Bruce protocol as described by Bamman et al. to determine fitness level [37]. Also, the upper limb body strength (UBS) and lower limb body strength (LBS) of participants were evaluated by one-repetition maximum (1RM) on the chest press and leg press machines, respectively, as previously described [34].

### Blood biochemistry

Fasting blood samples (~10 mL) were collected from the antecubital vein using standard procedures approximately 48 h before and after the last training session. Following the completion of blood sampling, the samples were centrifuged at 3000 rpm for 10 minutes, and serum was stored at -80°C until further analysis. Serum activin A (kit: Cusabio Co, sensitivity: 3.9 pg/ml), GDF15 (kit: Cusabio Co, sensitivity: 1.95 pg/ml), TGFβ1 (kit: Cusabio Co, sensitivity: 0.747 ng/ml), BDNF (kit: Abcam Co, sensitivity: 2.4 pg/ml), and irisin (kit: Cusabio Co, sensitivity: 0.78 ng/ml) concentrations were measured using commercial human ELISA kits. The intra- and inter-assay coefficients for all factors were < 8% and < 10%, respectively.

### Nutrient intake and dietary analysis

Participants were asked to maintain their habitual diet during the study. To minimize dietary variability, participants submitted 3-day (2 weekdays and 1 weekend) food records at baseline and 12 weeks of the intervention. Each item of food was individually entered into Diet Analysis Plus version 10 (Cengage, Boston, MA, USA), and total energy consumption and the amount of energy derived from proteins, fats, and carbohydrates were evaluated [38].

### Statistical analysis

Estimation of an appropriate sample size was conducted using the G*Power analysis software. Our rationale for sample size was based on previous studies [30, 34]. The analysis revealed that a sample size of at least 26 participants (n = 13 per group) was needed to provide power (1- β) of 0.80 (α = 0.05). The normality of data distribution was evaluated by the Shapiro–Wilk test. Analysis of variance (ANOVA) was applied to examine the intra- and inter-group differences. In the case of significant differences, a Fisher LSD post hoc test was used. Nutrition data were analysed using repeated measures ANOVA analysis. Statistical significance was set at P ≤ 0.05. All statistical procedures were analysed using statistical package for social sciences IBM SPSS Statistics 22 (IBM Software Group, Chicago, IL, USA) and were expressed as mean ± SD. All the figures were prepared in GraphPad Prism (version 8.0.2).

## RESULTS

### Dietary intake monitoring and compliance with exercise training and supplementation interventions

Overall adherence to both exercise training and supplementation was 100% across PR and SR. There were no reports of an adverse event from either nutritional intervention or the resistance training programme. The mean demographic characteristics of the two groups are summarized in Table 2. Nutrient analysis of the dietary records of PR and SR groups before and after the intervention period by using repeated measures ANOVA analysis is presented in Table 3. There were no significant differences in total energy intake or protein, fat, and carbohydrate intakes between the study groups (P > 0.05).

**TABLE 2:** Demographic data of participants

Variable	PR group (n = 15)	SR group (n = 15)
Age (year)	66.33.±.0.86	64.40.±.0.87
Height (cm)	167.93.±.0.92	168.±.0.97
Weight (kg)	64.85.±.0.96	61.09.±.0.98

Abbreviation: Values are presented as Mean ± SD; SR: soy milk + resistance training; PR: placebo + resistance training.

**TABLE 3:** Energy and macronutrients at the before and at the end of week 12

Variables	Group	Baseline	12 weeks	P-value
Energy (kcal/day)	PR	1773.33.±.49.77	1792.80.±.56.85	0.262
	SR	1762.00.±.50.11	1789.60.±.53.01	0.095

Protein (g/day)	PR	80.40.±.7.47	77.80.±.4.72	0.184
	SR	77.13.±.4.76	77.86.±.5.52	0.593

Carbohydrate (g/day)	PR	242.93.±.6.95	246.80.±.6.85	0.129
	SR	244.26.±.9.16	247.73.±.6.00	0.095

Fat (g/day)	PR	53.33.±.2.76	53.60.±.3.71	0.830
	SR	52.93.±.3.19	54.13.±.3.73	0.440

Abbreviation: SR: soy milk + resistance training; PR: placebo + resistance training. Values are Mean ± SD.

### Body composition

**FIG. 3:**
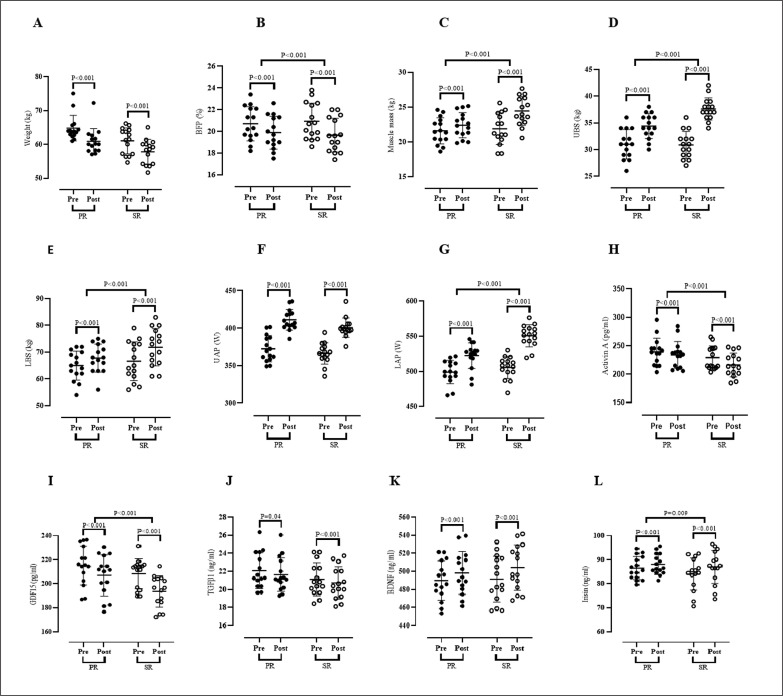
Changes in blood markers, physical performance and body compositions parameters in response to 12 weeks of soy milk ingestion immediately after resistance training. Weight, (A); BFP, Body Fat Percentage, (B); Muscle Mass, (C); UBS, Upper Body Strength (D); LBS, Lower Body Strength, (E); UAP, Upper Anaerobic Power, (F), LAP, Lower Anaerobic Power, (G), Activin A, (H); GDF15 (I); TGFβ1 (J); BDNF (K); and irisin (L) from pre-training to post-training in the PR (placebo + resistance training) and SR (soy milk + resistance training) groups. Error bars represent standard error of the mean.

Figure 3 presents values for body composition. Results of the present study indicated a significant decrease in weight [PR = -3.91 kg (95% confidence interval (CI), 5.55 to 2.27) (*P < 0.001*) and SR = -3.23 kg (95% CI 3.78 to 2.67) (*P < 0.001*) (Figure A)] and BFP [PR = -0.82% (95% CI, 0.94 to 0.69) (*P < 0.001*) and SR = -1.27% (95% CI 1.41 to 1.13) (*P < 0.001*) (Figure B)] over time. Also, a significant enhancement was experienced for muscle mass [PR = 0.81% (95% CI, -0.65 to -0.97) (*P < 0.001*) and SR = -2.56% (95% CI -2.22 to -2.89) (*P < 0.001*) (Figure C)] over time. ANOVA did not show that soy milk had a significant effect on body weight values (F_(1, 28)_ = 0.710, p = 0.407, η^2^ = 0.025) in interaction with the resistance training programme (Figure A). Only a similar effect of the resistance training programme was confirmed for both groups (F_(1, 28)_ = 78.387, p < 0.001, η^2^ = 0.736). A significant effect of using soy milk was observed in interaction with the resistance training programme for BFP (F_(1, 28)_ = 27.431, p < 0.001, η^2^ = 0.495) and muscle mass (F_(1, 28)_ = 99.631, p < 0.001, η^2^ = 0.781) (Figure B and C; respectively). Fisher’s LSD post-hoc test did not confirm that, either before or after 12 weeks of resistance training, the BFP values were statistically different between PR and SR groups (p > 0.05). Fisher’s LSD post-hoc test showed statistically significantly higher muscle mass values in the SR group compared to the PR group after the completion of the resistance training programme (p < 0.001).

### Physical fitness assessments

Figure 3 presents values for physical fitness assessments. Findings of the present study showed significantly increased UBS [PR = 3.40 kg (95% CI, -2.81 to -3.98) (*P < 0.001*) and SR = 6.73 kg (95% CI -6.20 to -7.26) (*P < 0.001*) (Figure D)], LBS [PR = 2.80 kg (95% CI, -1.79 to -3.80) (*P < 0.001*) and SR = 5.26 kg (95% CI – 4.31 to – 6.21) (*P < 0.001*) (Figure E)], UAP [PR = 34.34 W (95% CI, -26.74 to – 41.93) (*P < 0.001*) and SR = 38.62 W (95% CI – 33.76 to – 43.47) (*P < 0.001*) (Figure F)], and LAP [PR = 23.28 W (95% CI, -19.86 to – 26.71) (*P < 0.001*) and SR = 45.28 W (95% CI – 39.50 to – 51.06) (*P < 0.001*) (Figure G)] over time. ANOVA showed that soy milk had a significant impact on UBS (F_(1, 28)_ = 41.97, p < 0.001, η^2^ = 0.744), LBS (F_(1, 28)_ = 14.631, p < 0.001, η^2^ = 0.343) and LAP (F_(1, 28)_ = 49.34, p < 0.001, η^2^ = 0.638) in interaction with the resistance training programme (Figure D, E, and G; respectively). For UAP only a similar effect of resistance training programme for both PR and SR groups was found (F_(1, 28)_ = 301.43, p < 0.001, η^2^ = 0.915) (Figure F). Fisher’s LSD post-hoc test showed statistically significantly higher UBS and LAP values in the SR group compared to the PR group after the completion of the resistance training programme (p < 0.0001). Fisher’s LSD post-hoc test did not confirm that, either before or after the 12 weeks of the resistance training programme, the LBS values differed significantly between PR and SR groups (p > 0.05).

### Blood markers

Figure 3 presents values for serum concentrations of muscular-related biomarkers. There were significant reductions in serum concentrations of activin A [PR = -5.14 pg/ml (95% CI, 7.76 to 2.52) (*P < 0.001*) and SR = -12.89 pg/ml (95% CI, 16.70 to 9.08) (*P < 0.001*) (Figure H)], GDF15 [PR = -8.15 pg/ml (95% CI, 10.84 to 5.45) (*P < 0.001*) and SR = -14.79 pg/ml (95% CI, 17.43 to 12.15) (*P < 0.001*) (Figure I)], and TGFβ1 [PR = -0.43 pg/ml (95% CI, 0.85 to 0.02) (*P = 0.04*) and SR = -0.41 pg/ml (95% CI, 0.48 to 0.33) (*P < 0.001*) (Figure J)], and significantly increased serum concentrations of BDNF [PR = 8.37 ng/ml (95% CI, -5.63 to -11.11) (*P < 0.001*) and SR = 12.77 ng/ml (95% CI, -8.72 to -16.81) (*P < 0.001*) (Figure K)], and Irisin [PR = 1.56 ng/ml (95% CI, -0.81 to -2.30) (*P < 0.001*) and SR = 2.94 ng/ml (95% CI, -2.34 to – 3.53) (*P < 0.001*) (Figure L)] over time. ANOVA showed that the usage of soy milk had a significant influence on activin A (F_(1, 28)_ = 12.910, p < 0.001, η^2^ = 0.316), GDF15 (F_(1, 28)_ = 14.232, p < 0.001, η^2^ = 0.337) and irisin (F_(1, 28)_ = 9.639, p = 0.004, η^2^ = 0.256) in interaction with the resistance training programme (Figure H, I, and L; respectively). Fisher’s LSD post-hoc test showed significantly higher values of activin A (p = 0.036) and GDF15 (p = 0.019) in the SR group compared to the PR group after completion of the resistance training programme. Fisher’s LSD post-hoc test did not confirm that neither before or after the 12 weeks of the resistance training programme the irisin levels differed significantly between the PR and SR groups (p > 0.05). For BDNF (F_(1, 28)_ = 86.23, p < 0.001, η^2^ = 0.757) and TGFβ1 (F_(1, 28)_ = 18.671, p < 0.001, η^2^ = 0.400) only a similar effect of the resistance training programme was observed for both PR and SR groups (Figure J).

## DISCUSSION

This was the first randomized controlled clinical trial to examine the effects of soy milk ingestion during a resistance training programme in older men. The main findings of our study were that in both SR and PR groups, significantly increased muscle mass and strength, and serum concentrations of BDNF and irisin, and decreased BFP, serum concentrations of activin A, GDF15, and TGFβ1 were observed. The results showed that soy milk ingestion (post-exercise) during 12 weeks of resistance training increased muscle mass, muscle performance (strength and power), and serum concentrations of irisin compared to a placebo; however, the same was not observed in serum concentrations of BDNF. Also, it was found that soy milk ingestion (post-exercise) during 12 weeks of resistance training decreased serum concentrations of activin A and GDF15, compared to a placebo; however, the same was not observed in serum concentrations of TGFβ1 and BFP. There were no adverse events reported from either nutritional intervention or the resistance training programme.

The significant gain in muscle mass and muscular strength from soy milk ingestion compared to placebo in older men was in agreement with the findings of the previous research. For instance, Chiang et al. reported that soy milk (200 mL) during 12 weeks of mild resistance training significantly increased muscle mass and handgrip strength in very old nursing home residents with sarcopenia [39]. Orsatti et al. found that adding soy to milk combined with 16 weeks of resistance training resulted in a more significant gain in muscle mass and strength [40]. Nabuco et al. observed that 12 weeks of whey protein (1 · 4 g/kg per day) along with resistance training led to a significant gain in muscle mass and strength and functional capacity in older women [3]. In addition, it was demonstrated that taking protein about 1 · 6 g/kg per day led to a significant gain in muscle mass and strength [41]. Resistance training stimulates rates of muscle protein synthesis (MPS), which are further improved by the presence of dietary proteins [42]. Based on a meta-analysis by Messina et al. [43], leucine, one of the three branched-chain amino acids, is able to stimulate the activation of MPS. In particular, leucine activates MPS through the mTOR complex 1 (mTORC1); therefore it prevents muscle atrophy during the aging process [43]. In the present study, it may be possible that an increase of MPS led to significant enhancement of muscular accretion and muscular strength during the intervention. However, MPS was not evaluated in the present study, but one of the possible reasons for an increase in muscle mass is MPS. Improvement of muscle mass and muscular strength after soy milk ingestion and resistance training might be due to the mTORC1 signalling cascade, which integrates signals from mechanical stimuli, growth factors, and nutrients to stimulate MPS. Therefore, the combination of soy milk and resistance training had beneficial effects on elevating muscle mass and muscular strength.

Muscle protein balance is also influenced by various hormones and myokines, which have been suggested to alter the balance between anabolic and catabolic stimuli in muscle, leading to an increase or decrease in muscle mass. Among TGFβ isoforms, TGFβ1 is the most abundant and ubiquitously expressed isoform, which has a key role in various biological processes, including cell growth, muscle differentiation, apoptosis, tissue development, and inflammation [44]. TGFβ1 is a potent inhibitor of growth and differentiation in myoblasts and vascular smooth muscle cells; it also suppresses division and blocks fusion of satellite cells by suppressing myogenic factors [44]. In addition, activin A and GDF-15, other members of the TGF-β family, seem to have an inverse relationship with skeletal muscle growth and differentiation [45]. We found a significant reduction in serum concentrations of TGFβ1, activin A, and GDF15 in both groups. However, participants in the SR group experienced the most significant reduction in serum concentrations of activin A and GDF15 compared with the PR group. The mechanism of the effect of soy milk and resistance training on serum concentrations of activin A and GDF15 is still unclear. Elevated expression of activins promotes muscle wasting and cachexia, whereas blocking of activin type II receptors protects skeletal muscle from atrophy and leads to strong hypertrophy [45]. Chen et al. [46] reported that enhancement of circulating activin A not only promoted the reduction of muscle mass and body weight in a dose-dependent manner but also reduced muscle function, highlighting the therapeutic potential of activin A inhibitors [46]. Therefore, it may have been expected that a significant reduction in serum concentration of activin A and GDF15 in the SR group may be due to a significant gain of muscle mass. In this regard, Bagheri et al. reported a modest correlation between the changes in activin A and lean mass following post-exercise Icelandic yogurt consumption in healthy untrained older men [42]. Soy milk ingestion during resistance training can control protein synthesis by intracellular signalling pathways [47]. Protein consumption made a positive balance, which led to the transfer of amino acids into the cell [31]. Potential mechanisms for these changes are the synergy and interaction of soy milk and resistance training. Nevertheless, the superiority of the interaction of soy milk supplementation and resistance training elucidates the improvement of skeletal muscle regulatory markers by suppressing activin A and GDF15 concentrations in skeletal muscle.

According to the literature, skeletal muscle is increasingly classified as an endocrine organ that can release a variety of signalling molecules and regulate cytokines called myokines, such as irisin, which regulates several aging-related physiological and pathological processes [22]. Previous research showed a positive correlation between concentrations of irisin and muscle mass gains in humans [22]. A recent study by Chang et al. [48] reported that a low blood concentration of irisin was a sensitive molecular marker for muscle weakness and atrophy [48]. Huh et al. [49] reported a positive correlation between the decrease in serum irisin concentration and the loss of muscle mass associated with aging [49]. Kim et al. [50] stated that there was a positive correlation between the changes in circulating level of irisin and the change of muscle mass following resistance training in obese adults [50]. The present study used 12 weeks of resistance training with soy milk supplementation, which led to a significant rise in serum concentrations of irisin in the SR compared to the PR group. These differences may be due to irisin responsiveness to muscle mass gain [22], since it appears to be upregulated in response to an increase in muscle mass, which was observed in the soy milk ingestion group. Considering the benefits of soy milk ingestion after resistance training in improving muscle strength and function for the frail elderly, soy milk ingestion after resistance training could seem to be a beneficial intervention strategy to increase irisin concentrations in the aging population. A growing body of studies has demonstrated that lower serum concentrations of BDNF are correlated with the aging process [51, 52]. The change in concentrations of BDNF with increasing age and neuronal loss in older persons has been shown to be related to low peripheral BDNF levels [51]. Our study indicated that both SR and PR groups experienced similar and significant increases in serum concentrations of BDNF. These results are contradictory to the findings of Gomes et al., who noted that serum concentrations of BDNF were increased in elderly men after chronic exercise (12 weeks of aerobic training, consisting of a 50-min walk 3 times per week) [53]. Goekint et al. reported an insignificant increase in serum concentrations of BDNF after eight weeks of resistance training in untrained subjects [54]. To the best of our knowledge, the present study was the first one to evaluate the effect of soy milk ingestion during resistance training on circulating BDNF in elderly men. Our results suggest that resistance training interventions provide sufficient exercise stimulus to increase concentrations of BDNF in elderly men. The effect of using soy milk in interaction with the resistance training programme was not proven, although the trend of BDNF change in the SR group was greater than in the PR group (though not statistically significant).

The strengths of this study are triple. Firstly, the study design is a randomized, controlled clinical trial. Secondly, we proved the benefits of soy milk ingestion and resistance training, and their direct effects on muscular-related biomarkers as well as fitness and body composition markers in older men. Thirdly, valid and objective measures were utilized for the evaluation of muscular-related biomarkers, body composition, and physical fitness. This study also had some limitations. One of the limitations of this study was the lack of dietary intake monitoring, and therefore dietary intake was not carefully controlled. However, given the uncertain effect of soy milk on the results of our study, further research is needed to develop more effective diets for this older population.

## CONCLUSIONS

In conclusion, the strategic ingestion of soy milk (post-exercise) during 12 weeks of resistance training augmented lean mass, strength, and power, and altered serum concentrations of skeletal muscle regulatory markers (activin A, GDF15) in older men compared to placebo. Hence, older men may use soy milk ingestion after exercise to improve muscular performance.

## References

[1] Danan G, Matthew ED. Encyclopedia of Gerontology and Population Aging. Springer International Publishing. 2019:29–1.

[2] Nabuco HCG, Tomeleri CM, Fernandes RR, Sugihara Junior P, Cavalcante EF, Cunha PM, Antunes M, Nunes JP, Venturini D, Barbosa DS, Burini RC, Silva AM, Sardinha LB, Cyrino ES. Effect of whey protein supplementation combined with resistance training on body composition, muscular strength, functional capacity, and plasma-metabolism biomarkers in older women with sarcopenic obesity: A randomized, double-blind, placebo-controlled trial. Clin Nutr ESPEN. 2019; 32:88–95. 31221297 10.1016/j.clnesp.2019.04.007

[3] Nabuco HCG, Tomeleri CM, Sugihara Junior P, Fernandes RR, Cavalcante EF, Antunes M, Ribeiro AS, Teixeira DC, Silva AM, Sardinha LB, Cyrino ES. Effects of Whey Protein Supplementation Pre- or Post-Resistance Training on Muscle Mass, Muscular Strength, and Functional Capacity in Pre-Conditioned Older Women: A Randomized Clinical Trial. Nutrients. 2018; 10(5):563. 29751507 10.3390/nu10050563 PMC5986443

[4] Hooshmand-Moghadam B, Eskandari M, Golestani F, Rezae S, Mahmoudi N, Gaeini AA. The effect of 12-week resistance exercise training on serum levels of cellular aging process parameters in elderly men. Exp Gerontol. 2020; 141:111090. 32919015 10.1016/j.exger.2020.111090

[5] Sakuma K, Yamaguchi A. Novel intriguing strategies attenuating to sarcopenia. J Aging Res. 2012; 2012:251217. 22500226 10.1155/2012/251217 PMC3303581

[6] Patel VK, Demontis F. GDF11/myostatin and aging. Aging (Albany NY). 2014; 6(5):351–2. 24902527 10.18632/aging.100666 PMC4069261

[7] Schiaffino S, Dyar KA, Ciciliot S, Blaauw B, Sandri M. Mechanisms regulating skeletal muscle growth and atrophy. FEBS J. 2013; 280(17):4294–314. 23517348 10.1111/febs.12253

[8] Xu X, Zheng L, Yuan Q, Zhen G, Crane JL, Zhou X, Cao X. Transforming growth factor-β in stem cells and tissue homeostasis. Bone Res. 2018; 6:2. 29423331 10.1038/s41413-017-0005-4 PMC5802812

[9] Ismaeel A, Kim JS, Kirk JS, Smith RS, Bohannon WT, Koutakis P. Role of Transforming Growth Factor-β in Skeletal Muscle Fibrosis: A Review. Int J Mol Sci. 2019; 20(10):2446. 31108916 10.3390/ijms20102446 PMC6566291

[10] Yatsuga S, Fujita Y, Ishii A, Fukumoto Y, Arahata H, Kakuma T, Kojima T, Ito M, Tanaka M, Saiki R, Koga Y. Growth differentiation factor 15 as a useful biomarker for mitochondrial disorders. Ann Neurol. 2015; 78(5):814–23. 26463265 10.1002/ana.24506 PMC5057301

[11] Barma M, Khan F, Price RJG, Donnan PT, Messow CM, Ford I, McConnachie A, Struthers AD, McMurdo MET, Witham MD. Association between GDF-15 levels and changes in vascular and physical function in older patients with hypertension. Aging Clin Exp Res. 2017; 29(5):1055–1059. 27734214 10.1007/s40520-016-0636-0 PMC5589783

[12] Reza MM, Subramaniyam N, Sim CM, Ge X, Sathiakumar D, McFarlane C, Sharma M, Kambadur R. Irisin is a pro-myogenic factor that induces skeletal muscle hypertrophy and rescues denervation-induced atrophy. Nat Commun. 2017; 8(1):1104. 29062100 10.1038/s41467-017-01131-0 PMC5653663

[13] Chang JS, Kong ID. Irisin prevents dexamethasone-induced atrophy in C2C12 myotubes. Pflugers Arch. 2020; 472(4):495–502. 32219531 10.1007/s00424-020-02367-4 PMC7165150

[14] Zhang Z, Wang B, Fei A. BDNF contributes to the skeletal muscle anti-atrophic effect of exercise training through AMPK-PGC1α signaling in heart failure mice. Arch Med Sci. 2019; 15(1):214–222. 30697273 10.5114/aoms.2018.81037 PMC6348347

[15] Planella-Farrugia C, Comas F, Sabater-Masdeu M, Moreno M, Moreno-Navarrete JM, Rovira O, Ricart W, Fernández-Real JM. Circulating Irisin and Myostatin as Markers of Muscle Strength and Physical Condition in Elderly Subjects. Front Physiol. 2019; 10:871. 31354522 10.3389/fphys.2019.00871 PMC6637304

[16] Sheida V, Fathi M, Mir E. The effect of core stabilization exercise on the serum level of Activin A and back performance scale in elderly women with chronic low back pain: a randomized clinical trial. Medical Sciences. 2020; 30(11):867–875.

[17] Fujita Y, Taniguchi Y, Shinkai S, Tanaka M, Ito M. Secreted growth differentiation factor 15 as a potential biomarker for mitochondrial dysfunctions in aging and age-related disorders. Geriatr Gerontol Int. 2016; 16 Suppl 1:17–29. 27018280 10.1111/ggi.12724

[18] Baccarelli A, Morpurgo PS, Corsi A, Vaghi I, Fanelli M, Cremonesi G, Vaninetti S, Beck-Peccoz P, Spada A. Activin A serum levels and aging of the pituitary-gonadal axis: a cross-sectional study in middle-aged and elderly healthy subjects. Exp Gerontol. 2001; 36(8):1403–12. 11602213 10.1016/s0531-5565(01)00117-6

[19] Krieger JW. Single vs. multiple sets of resistance exercise for muscle hypertrophy: a meta-analysis. J Strength Cond Res. 2010; 24(4):1150–9. 20300012 10.1519/JSC.0b013e3181d4d436

[20] Grgic J, Garofolini A, Orazem J, Sabol F, Schoenfeld BJ, Pedisic Z. Effects of Resistance Training on Muscle Size and Strength in Very Elderly Adults: A Systematic Review and Meta-Analysis of Randomized Controlled Trials. Sports Med. 2020; 50(11):1983–1999. 32740889 10.1007/s40279-020-01331-7

[21] Máderová D, Krumpolec P, Slobodová L, Schön M, Tirpáková V, Kovaničová Z, Klepochová R, Vajda M, Šutovský S, Cvečka J, Valkovič L, Turčáni P, Krššák M, Sedliak M, Tsai CL, Ukropcová B, Ukropec J. Acute and regular exercise distinctly modulate serum, plasma and skeletal muscle BDNF in the elderly. Neuropeptides. 2019; 78:101961. 31506171 10.1016/j.npep.2019.101961

[22] Kim HJ, So B, Choi M, Kang D, Song W. Resistance exercise training increases the expression of irisin concomitant with improvement of muscle function in aging mice and humans. Exp Gerontol. 2015; 70:11–7. 26183690 10.1016/j.exger.2015.07.006

[23] Guimarães-Ferreira L, Cholewa JM, Naimo MA, Zhi XI, Magagnin D, de Sá RB, Streck EL, Teixeira Tda S, Zanchi NE. Synergistic effects of resistance training and protein intake: practical aspects. Nutrition. 2014; 30(10):1097–103. 24751198 10.1016/j.nut.2013.12.017

[24] Moro T, Brightwell CR, Velarde B, Fry CS, Nakayama K, Sanbongi C, Volpi E, Rasmussen BB. Whey Protein Hydrolysate Increases Amino Acid Uptake, mTORC1 Signaling, and Protein Synthesis in Skeletal Muscle of Healthy Young Men in a Randomized Crossover Trial. J Nutr. 2019; 149(7):1149–1158. 31095313 10.1093/jn/nxz053 PMC7443767

[25] McCroskery S, Thomas M, Maxwell L, Sharma M, Kambadur R. Myostatin negatively regulates satellite cell activation and self-renewal. J Cell Biol. 2003; 162(6):1135–47. 12963705 10.1083/jcb.200207056 PMC2172861

[26] Phillips SM, Tang JE, Moore DR. The role of milk- and soy-based protein in support of muscle protein synthesis and muscle protein accretion in young and elderly persons. J Am Coll Nutr. 2009; 28(4):343–54. 20368372 10.1080/07315724.2009.10718096

[27] Sethi S, Tyagi SK, Anurag RK. Plant-based milk alternatives an emerging segment of functional beverages: a review. J Food Sci Technol. 2016; 53(9):3408–3423. 27777447 10.1007/s13197-016-2328-3 PMC5069255

[28] Mohammad-Shahi M, Mowla K, Haidari F, Zarei M, Choghakhori R. Soy milk consumption, markers of inflammation and oxidative stress in women with rheumatoid arthritis: A randomised cross-over clinical trial. Nutrition & Dietetics. 2015; 73(2):139–145.

[29] Keshavarz SA, Nourieh Z, Attar MJ, Azadbakht L. Effect of Soymilk Consumption on Waist Circumference and Cardiovascular Risks among Overweight and Obese Female Adults. Int J Prev Med. 2012; 3(11):798–805. 23189232 PMC3506092

[30] Liao YH, Chen CN, Hu CY, Tsai SC, Kuo YC. Soymilk ingestion immediately after therapeutic exercise enhances rehabilitation outcomes in chronic stroke patients: A randomized controlled trial. NeuroRehabilitation. 2019; 44(2):217–229. 30856124 10.3233/NRE-182574

[31] Pourabbas M, Bagheri R, Hooshmand Moghadam B, Willoughby DS, Candow DG, Elliott BT, Forbes SC, Ashtary-Larky D, Eskandari M, Wong A, Dutheil F. Strategic Ingestion of High-Protein Dairy Milk during a Resistance Training Program Increases Lean Mass, Strength, and Power in Trained Young Males. Nutrients. 2021; 13(3):948. 33804259 10.3390/nu13030948 PMC7999866

[32] Eslami O, Shidfar F, Maleki Z, Jazayeri S, Hosseini AF, Agah S, Ardiyani F. Effect of Soy Milk on Metabolic Status of Patients with Nonalcoholic Fatty Liver Disease: A Randomized Clinical Trial. J Am Coll Nutr. 2019; 38(1):51–58. 30028245 10.1080/07315724.2018.1479990

[33] Calvani R, Marini F, Cesari M, Tosato M, Picca A, Anker SD, von Haehling S, Miller RR, Bernabei R, Landi F, Marzetti E; SPRINTT Consortium. Biomarkers for physical frailty and sarcopenia. Aging Clin Exp Res. 2017; 29(1):29–34. 10.1007/s40520-016-0708-1 28155180

[34] Bagheri R, Moghadam BH, Church DD, Tinsley GM, Eskandari M, Moghadam BH, Motevalli MS, Baker JS, Robergs RA, Wong A. The effects of concurrent training order on body composition and serum concentrations of follistatin, myostatin and GDF11 in sarcopenic elderly men. Exp Gerontol. 2020; 133:110869. 32035222 10.1016/j.exger.2020.110869

[35] Abreu EL, Cheng AL, Kelly PJ, Chertoff K, Brotto L, Griffith E, Kinder G, Uridge T, Zachow R, Brotto M. Skeletal muscle troponin as a novel biomarker to enhance assessment of the impact of strength training on fall prevention in the older adults. Nurs Res. 2014; 63(2):75–82. 24589644 10.1097/NNR.0000000000000018

[36] Ahmad A, Alghamdi SS, Mahmood K, Afzal M. Fenugreek a multipurpose crop: Potentialities and improvements. Saudi J Biol Sci. 2016; 23(2):300–10. 27307778 10.1016/j.sjbs.2015.09.015 PMC4894452

[37] Bamman MM, Hill VJ, Adams GR, Haddad F, Wetzstein CJ, Gower BA, Ahmed A, Hunter GR. Gender differences in resistance-training-induced myofiber hypertrophy among older adults. J Gerontol A Biol Sci Med Sci. 2003; 58(2):108–16. 12586847 10.1093/gerona/58.2.b108

[38] Eskandari M, Hooshmand Moghadam B, Bagheri R, Ashtary-Larky D, Eskandari E, Nordvall M, Dutheil F, Wong A. Effects of Interval Jump Rope Exercise Combined with Dark Chocolate Supplementation on Inflammatory Adipokine, Cytokine Concentrations, and Body Composition in Obese Adolescent Boys. Nutrients. 2020; 12(10):3011. 33007981 10.3390/nu12103011 PMC7600985

[39] Chiang FY, Chen JR, Lee WJ, Yang SC. Effects of Milk or Soy Milk Combined with Mild Resistance Exercise on the Muscle Mass and Muscle Strength in Very Old Nursing Home Residents with Sarcopenia. Foods. 2021; 10(11):2581. 34828861 10.3390/foods10112581 PMC8623877

[40] Orsatti FL, Maestá N, de Oliveira EP, Nahas Neto J, Burini RC, Nunes PRP, Souza AP, Martins FM, Nahas EP. Adding Soy Protein to Milk Enhances the Effect of Resistance Training on Muscle Strength in Postmenopausal Women. J Diet Suppl. 2018; 15(2):140–152. 28604135 10.1080/19390211.2017.1330794

[41] Hevia-Larraín V, Gualano B, Longobardi I, Gil S, Fernandes AL, Costa LAR, Pereira RMR, Artioli GG, Phillips SM, Roschel H. High-Protein Plant-Based Diet Versus a Protein-Matched Omnivorous Diet to Support Resistance Training Adaptations: A Comparison Between Habitual Vegans and Omnivores. Sports Med. 2021; 51(6):1317–1330. 33599941 10.1007/s40279-021-01434-9

[42] Bagheri R, Hooshmand Moghadam B, Candow DG, Elliott BT, Wong A, Ashtary-Larky D, Forbes SC, Rashidlamir A. Effects of Icelandic yogurt consumption and resistance training in healthy untrained older males. Br J Nutr. 2022; 127(9):1334–1342. 34121642 10.1017/S0007114521002166

[43] Messina M, Lynch H, Dickinson JM, Reed KE. No Difference Between the Effects of Supplementing With Soy Protein Versus Animal Protein on Gains in Muscle Mass and Strength in Response to Resistance Exercise. Int J Sport Nutr Exerc Metab. 2018; 28(6):674–685. 29722584 10.1123/ijsnem.2018-0071

[44] Nikooie R, Jafari-Sardoie S, Sheibani V, Nejadvaziri Chatroudi A. Resistance training-induced muscle hypertrophy is mediated by TGF-1-Smad signaling pathway in male Wistar rats. J Cell Physiol. 2020; 235(7–8):5649–5665. 31960436 10.1002/jcp.29497

[45] Oesen S, Halper B, Hofmann M, Jandrasits W, Franzke B, Strasser EM, Graf A, Tschan H, Bachl N, Quittan M, Wagner KH, Wessner B. Effects of elastic band resistance training and nutritional supplementation on physical performance of institutionalised elderly--A randomized controlled trial. Exp Gerontol. 2015; 72:99–108. 26341720 10.1016/j.exger.2015.08.013

[46] Chen JL, Walton KL, Winbanks CE, Murphy KT, Thomson RE, Makanji Y, Qian H, Lynch GS, Harrison CA, Gregorevic P. Elevated expression of activins promotes muscle wasting and cachexia. FASEB J. 2014; 28(4):1711–23. 24378873 10.1096/fj.13-245894

[47] Volpi E, Kobayashi H, Sheffield-Moore M, Mittendorfer B, Wolfe RR. Essential amino acids are primarily responsible for the amino acid stimulation of muscle protein anabolism in healthy elderly adults. Am J Clin Nutr. 2003; 78(2):250–8. 12885705 10.1093/ajcn/78.2.250 PMC3192452

[48] Chang JS, Kim TH, Nguyen TT, Park KS, Kim N, Kong ID. Circulating irisin levels as a predictive biomarker for sarcopenia: A cross-sectional community-based study. Geriatr Gerontol Int. 2017; 17(11):2266–2273. 28394089 10.1111/ggi.13030

[49] Huh JY, Panagiotou G, Mougios V, Brinkoetter M, Vamvini MT, Schneider BE, Mantzoros CS. FNDC5 and irisin in humans: I. Predictors of circulating concentrations in serum and plasma and II. mRNA expression and circulating concentrations in response to weight loss and exercise. Metabolism. 2012; 61(12):1725–38. 23018146 10.1016/j.metabol.2012.09.002 PMC3614417

[50] Kim HJ, Lee HJ, So B, Son JS, Yoon D, Song W. Effect of aerobic training and resistance training on circulating irisin level and their association with change of body composition in overweight/obese adults: a pilot study. Physiol Res. 2016; 65(2):271–9. 26447516 10.33549/physiolres.932997

[51] Forti LN, Van Roie E, Njemini R, Coudyzer W, Beyer I, Delecluse C, Bautmans I. Dose-and gender-specific effects of resistance training on circulating levels of brain derived neurotrophic factor (BDNF) in community-dwelling older adults. Exp Gerontol. 2015; 70:144–9. 26297701 10.1016/j.exger.2015.08.004

[52] Walsh JJ, Scribbans TD, Bentley RF, Kellawan JM, Gurd B, Tschakovsky ME. Neurotrophic growth factor responses to lower body resistance training in older adults. Appl Physiol Nutr Metab. 2016; 41(3):315–23. 26886517 10.1139/apnm-2015-0410

[53] Gomes WF, Lacerda AC, Mendonça VA, Arrieiro AN, Fonseca SF, Amorim MR, Teixeira AL, Teixeira MM, Miranda AS, Coimbra CC, Brito-Melo GE. Effect of exercise on the plasma BDNF levels in elderly women with knee osteoarthritis. Rheumatol Int. 2014; 34(6):841–6. 23739920 10.1007/s00296-013-2786-0

[54] Goekint M, De Pauw K, Roelands B, Njemini R, Bautmans I, Mets T, Meeusen R. Strength training does not influence serum brain-derived neurotrophic factor. Eur J Appl Physiol. 2010; 110(2):285–93. 20467874 10.1007/s00421-010-1461-3

